# Important Post‐Harvest Characteristics of Some Kiwifruits Dried Using Innovative Technology

**DOI:** 10.1002/fsn3.72128

**Published:** 2026-07-16

**Authors:** Z. Göksel, A. Atak, K. A. Kahraman, C. Tunçkal

**Affiliations:** ^1^ Department of Food Technology Atatürk Horticultural Central Research Institute Yalova Türkiye; ^2^ Department of Horticulture, Agriculture Faculty Bursa Uludağ University Bursa Türkiye; ^3^ Atatürk Horticultural Central Research Institute Yalova Türkiye; ^4^ Electric and Energy Department, Yalova Community College Yalova University Yalova Türkiye

**Keywords:** breeding, color, dried kiwifruits, sensory analyses, yellow flesh

## Abstract

Kiwifruit production is increasing every year in Türkiye and worldwide. Along with this increase, processing kiwifruit in various ways, in addition to fresh consumption, significantly increases overall consumption and adds value. In this study, three new kiwifruit varieties (İlkaltın, HO8 and J284) developed within the scope of the only kiwifruit breeding program carried out in Türkiye were dried in a heat pump drying system, and their important quality characteristics and shelf life were compared. Previous studies have reported that heat pump drying technology is an energy‐efficient drying method, which was used in this study to dry kiwifruit. The product's moisture content and the drying air temperature were controlled using sensors integrated into the system. After the yellow fruit‐fleshed kiwifruit varieties reached the desired water values in the heat pump dryer, color, pH, acidity, water‐soluble solids, L‐Ascorbic acid, total sugar, total antioxidant activity, total phenolic contents, microbiological activity and sensory analyses were performed. It was determined that these new yellow fruit‐fleshed kiwifruits, which were especially dried, were attractive in terms of consumability and taste–aroma‐appearance. The dried yellow‐fleshed kiwifruit cultivars received high sensory scores from the panelists, indicating good consumer acceptability. The most prominent quality characteristics are their ability to maintain their bright, vibrant yellow color after drying and their higher sugar and lower acid content. The results obtained showed that the new kiwifruit varieties are quite suitable for consumption, not only fresh but also dried. In addition, not only small fruits (under 80–90 g) but also normal‐sized fruits can be sold at higher prices and provide more profit to their growers if they are dried in addition to fresh consumption.

## Graphical abstract

Three new kiwi varieties (İlkaltın, HO8, and J284), developed within the scope of the only kiwifruit breeding program carried out in Türkiye, were dried in a heat pump drying system, and their important quality characteristics and shelf life were compared. The product's moisture content and the drying air temperature were controlled using sensors integrated into the system. After the yellow‐fruited kiwifruit varieties were reduced to the desired water content in the heat pump dryer, they were comprehensively evaluated for color, pH, acidity, water‐soluble solids, L‐ascorbic acid, total sugar, total antioxidant activity, total phenolic content, microbiological activity, and sensory analysis.

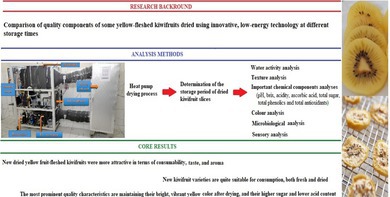

## 1 Introduction

Globally, 4,433,000 tons of kiwifruit are produced on a total area of 286,348 ha. There is an increase of approximately 1 million tons compared to 10 years ago. In Türkiye, there is a production of nearly 90,000 tons of kiwifruit, more than double the production compared to 10 years ago (FAOSTAT 2023). Türkiye ranks seventh in the world with this production figure, and it is expected to rise to even higher ranks, especially with the increase in production of newly developed kiwifruit varieties. With increasing production, different processing methods that can be an alternative to fresh consumption are gaining importance. Kiwifruit is a type of fruit that attracts consumers' attention due to its taste and high nutritional quality. Kiwifruit has become a popular fruit today due to its different flesh colors, high vitamin C content, richness in a wide variety of phytonutrients such as carotenoids, lutein, phenolics, flavonoids, and chlorophyll. It has many beneficial components for health and has a strong potential for industrial production (Kaya et al. 2010; Henare et al. 2012; Asadi, Ghasemnezhad, Olfati, et al. 2024). In addition, yellow and red fleshed varieties developed due to breeding studies in recent years are preferred more with their higher sugar and lower acid content and unique aromas (Asadi, Ghasemnezhad, Bakhshipour, et al. 2024; Ghasemnezhad et al. 2022; Kahraman et al. 2022). However, when kiwi fruits are dried by different methods, some of the important quality components may be lost at varying rates (Bhat et al. 2022). Many of the kiwifruits, which have a climacteric structure, especially the varieties of the *Actinidia chinensis* species, can be cold stored for a shorter period of time and have a limited shelf life when ripe (Meena et al. 2018). Therefore, in order to extend the shelf life and commercial sale period of kiwifruit, the product range needs to be increased by using different processing methods (Guroo et al. 2017).

Most kiwifruit species and varieties have a very short shelf life, and even under refrigerated conditions, softening and vitamin loss limit their widespread consumption (Maskan 2001). In order to extend the shelf life of kiwifruits, moisture needs to be reduced in the fruit (Tian et al. 2015).

Drying is one of the most preferred methods used to preserve the quality characteristics of many fruit species for a long time. Dried fruits are a healthy snack that have been increasingly consumed in recent years due to their rich bioactive compounds and their ability to be stored for long periods without spoiling (Ishiwata et al. 2004). Many dried fruits are an important part of diets in many countries due to their unique aromas and varying unique textures (Atak et al. 2022).

When high efficiency is required during dried fruit production, mechanical drying, which is safe, fast and more controllable, is generally preferred (Arslan and Alibaş 2024).

Important quality parameters of dried kiwifruits may vary according to the species and varieties Yuan et al. (2023). Therefore, many important components of new varieties should be analyzed after drying and clarified, especially for the consumers. In addition, the shelf life of dried products is also important, and their long‐term storage is an important characteristic desired by both the sellers of these products and the consumers (El‐Ramady et al. 2015).

At the same time, the value‐added products derived from these newly cultivated varieties are attracting consumer attention across markets due to their nutritional content and key advantages (Guo et al. 2024).

Although the traditional hot air drying method is the most common drying method, the most important disadvantages of this method are its high energy consumption and the need for high drying temperatures. Different drying methods, such as hot air and oven drying, freeze drying, microwave and infrared drying, and vacuum drying, all have higher energy requirements. Studies have been conducted on alternative drying technologies to eliminate these disadvantages. One of these is the heat pump drying technology, which has low energy consumption. This drying method consumes 60%–80% less energy than traditional dryers at the same temperatures (Strommen et al. 2002). In addition, the moisture content of the product and the drying air temperature can be easily controlled with the help of sensors integrated into the system. Heat pump drying was selected because previous studies have reported its lower energy consumption and higher energy efficiency compared with conventional drying systems. However, this study focuses on the quality and shelf life characteristics of dried fruits instead of energy performance parameters. The most important characteristics of this study are the comprehensive evaluation of yellow‐fleshed kiwi varieties obtained from newly developed Turkish kiwi breeding programs after drying with a heat pump. Instead of focusing on the drying technology itself, the study aims to determine the suitability of these varieties for dried fruit production by integrating assessments of nutritional quality, bioactive compounds, sensory properties, and storage stability. In this study, detailed evaluations were made regarding both important quality components and the shelf life of three kiwifruit varieties, a newly bred line, after drying in a heat pump dryer.

## 2 Materials and Methods

### 2.1 Plant Materials

**FIGURE 1:**
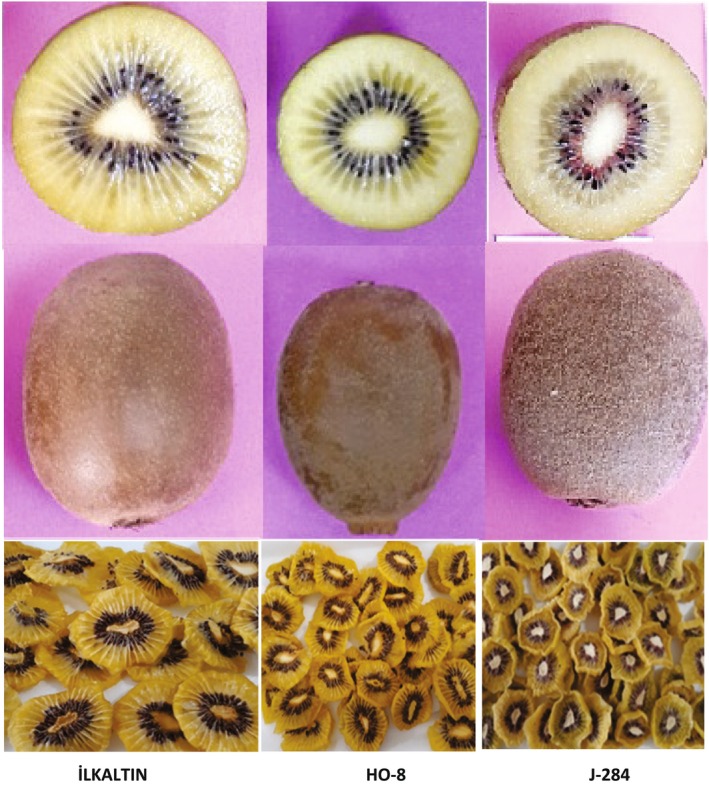
Fresh and dried photos of yellow‐fleshed kiwifruit varieties.

Three kiwifruit varieties developed by Yalova Atatürk Horticultural Central Research Institute were used in this study. These varieties are İlk Altın, HO8, and J284 kiwifruit varieties, all of which have yellow fruit flesh, belonging to the *Actinidia chinensis* species (Figure 1).

Kiwifruits were harvested when the flesh color turned yellow and reached a minimum brix value of 7. Then, the fruits were kept in cold storage for a while and then taken to dry when they reached the desired maturity values. When the TSS value reached 12% Brix and 3 N hardness values for maturity, they were taken out of storage, and the drying process was started without any pretreatment.

### 2.2 Preparation of Kiwifruit Samples

Before the kiwifruit varieties were processed, they were selected to be of standard size (80–90 g). Then, the kiwifruits were peeled. The fruits were sliced with a circular slicer with a thickness of 9 mm, which was determined as the most suitable slice thickness for kiwifruit by preliminary trials.

### 2.3 Drying Process With Heat Pump

**FIGURE 2:**
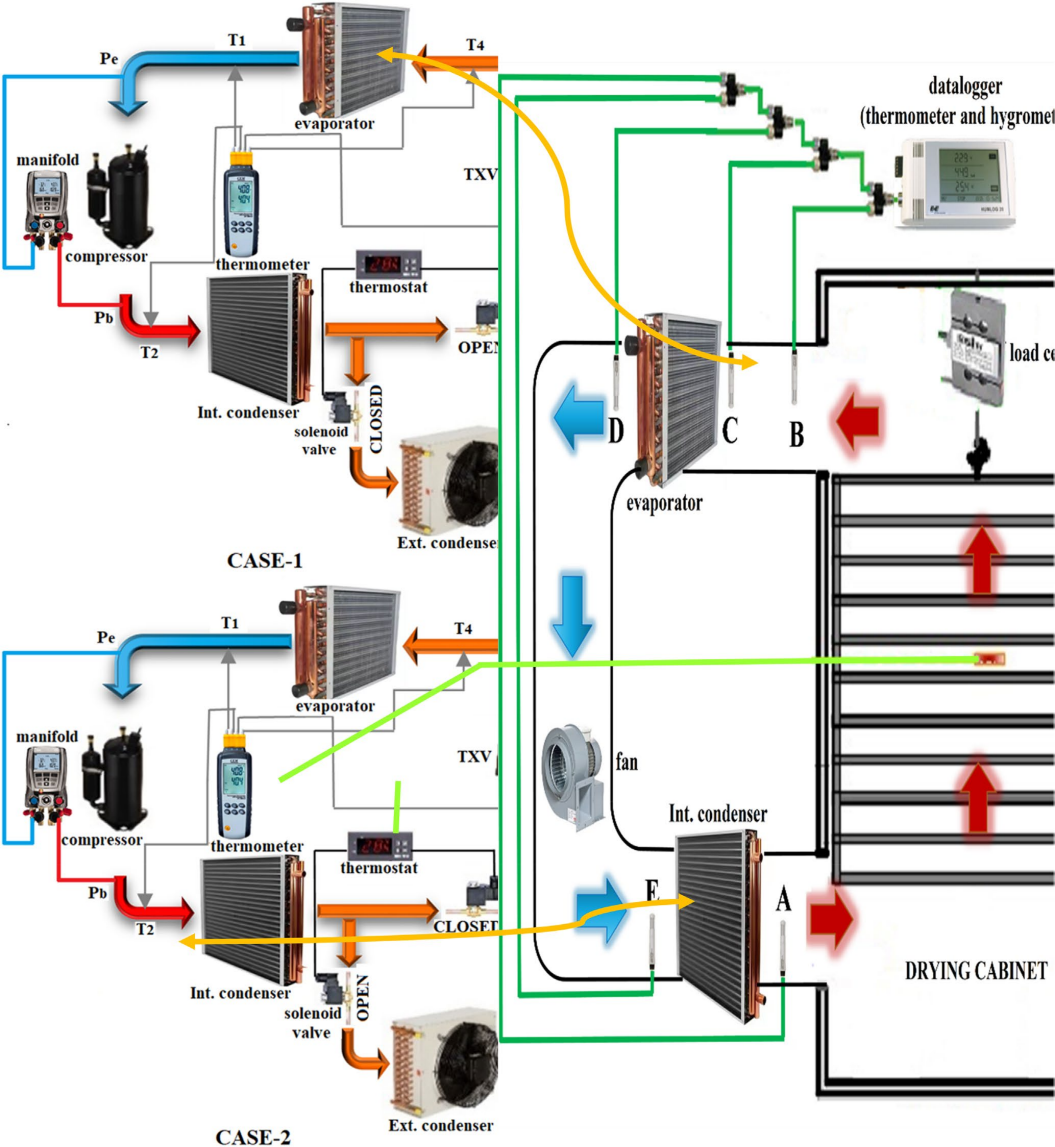
A detailed diagram showing how the drying process is carried out with the heat pump drying system. (The heat pump drying system provides control with a digital thermostat according to the desired drying temperature. The operation of the heat pump drying system in the refrigerant line is shown in the “CASE‐1” and “CASE‐2” states).

The drying system we used in our study, which operates with a heat pump method, was chosen due to some important advantages (Figure 2). The most important of these are drying time and energy consumption. During the drying process, the temperature was set to 45°C, while the air movement inside was adjusted to a speed of 1 m/s (Tunçkal et al. 2016). During the drying process, the final water content of the kiwifruit slices was continuously monitored, and the drying process was continued until it was reduced to the desired levels. 15%. All kiwifruit varieties were dried under the same drying conditions. The fruits were sliced to approximately 9 mm thick and dried until the final moisture content reached max.15%. During drying, care was taken to place the products in a single layer on the stainless steel trays, and the same loading conditions were applied to all varieties. Thus, the aim was to compare the quality differences between the varieties while keeping the effect of the drying parameters constant. Thirty kiwifruit slices were then randomly selected, and these slices were divided into three groups of ten and analyzed in three replications.

### 2.4 Determination of the Storage Period of Dried Kiwifruit Slices

Dried kiwifruit slices were stored at room temperature for 12 months to determine their shelf life. During the storage period, samples were collected every 3 months and analyzed. The analyses examined water activity, total mold and yeast content, shelf life, and color of the dried products. Maintaining a water activity below 0.60 in dried fruits significantly inhibits microbial growth, while the use of oxygen barrier packaging reduces color loss, ascorbic acid degradation, and oxidative browning. Therefore, storing dried yellow kiwifruit in vacuum packaging with low water activity and high barrier properties significantly extends its shelf life. Dried yellow kiwifruit samples were packaged using heat‐sealed laminated 100 mg barrier bags (PE) that provide high resistance to oxygen and moisture transfer. After packaging, the samples were stored for 12 months at 24°C ± 2°C and 50% ± 5% relative humidity in a dark environment. Exposure to direct sunlight was avoided during the storage period. Analyses were performed at 0, 3, 6, 9, and 12 months using independently stored packages. Three packages were randomly selected in each sampling period, and destructive sampling was applied.

### 2.5 Water Activity Analysis

With the Novasina water activity device, sorption isotherms were also determined (measurement accuracy ± 0.001 when aw value is 0–1). The dried kiwifruit is grinded homogeneously or cut into small pieces. The sample is kept at room temperature. The sample is placed in the sample chamber of the instrument. The instrument determines the water activity by measuring the water vapor pressure of the sample. It is important that the sample comes to room temperature before the measurement. The measurement result is read directly on the instrument display. The fresh water content values of the kiwifruit varieties ranged from 0.96 to 0.99 aw. After drying, these values decreased to 0.50–0.55 aw.

### 2.6 Color Analysis

Color analysis of dried kiwifruit slices was performed using a CIE Minolta chromometer (Minolta, CR‐300, Japan). L* (brightness), a* (+red‐green), b* (yellow‐blue) were measured in different parts of dried kiwifruit slices. The Minolta Chromameter used for the measurements was calibrated with a standard white plate before use. L* indicates the lightness‐darkness of the color. The L* value reaches its maximum value as it approaches 100 and is based on the reflection of 100% of the light sent to the surface. The a* value indicates the color change from green to red, and the b* value indicates the color change from yellow to blue. Increasingly negative or positive values indicate darkening of the color. Chroma and Hue° values were calculated using the a* and b* values.

### 2.7 pH

To measure the pH of kiwifruit slices, 10 g of fresh and 10 g of dried kiwifruit samples were taken. Pure water was added to these samples until a total of 250 mL was obtained. The mixture was then blended for 120 s. The pH of the homogenized mixture was then measured using a digital pH meter (Consort 514).

### 2.8 The Amount of Water‐Soluble Solids (Brix) and Acidity

Atago Digital hand refractometer (Atago Pal‐Bx|Acid8, Japan) was used to determine the acidity and Brix values of fresh kiwifruits. The amount of water‐soluble dry matter (brix) was determined as % by extracting 3 replicates of fruit juice from fresh kiwifruit samples at room temperature. Similarly, for acidity analyses, 1 mL of fruit juice was dropped into a beaker and this diluted solution was determined by dropping it onto the refractometer after completing it with 50 mL of pure water.

### 2.9 Texture Analysis of Samples

The Shimadzu EZ‐LX Long Stroke model device was used to perform texture analysis of kiwifruit slices. The firmness values of the samples were measured in terms of maximum force (N) using this device. This analysis is based on the force–deformation characteristics of the fruit, and evaluation of the rheological properties of kiwifruit is the measure of the maximal force or stress needed to perforate the fruit, generally called Magness–Taylor firmness. This firmness is usually performed on peeled fruits, so we tested peeled fruits.

In order to do the rheological tests, samples were placed in their natural rest position, and analyses were accomplished in the orientation of thickness. The texture analyzer was fitted with a flat probe (7 mm diameter) which was forced into the fruit at constant speed (10 mm/min for peeled fruits) to an 8 mm depth for peeled samples.

### 2.10 Determination of L‐Ascorbic Acid

The L‐Ascorbic acid (LAA) content (mg/100 g fresh weight/dry weight) was determined by using a reflectometer (RQ‐flex‐plus, Merck, Japan) as described by Orikasa et al. (2008 and 2014). A 5 g portion of the sample was placed in a beaker, and approximately 100 mL of 1% metaphosphoric acid solution was added. The mixture was homogenized for 1 min and then centrifuged at 8000 rpm for 10 min. The supernatant (Table 1)solution was used to analyze the ascorbic acid concentration. The total quantity of LAA (mg) in the test sample was calculated by multiplying the measured content (mg/100 g fresh weight/dry weight) by the initial sample weight (g).

**TABLE 1:** Some quality characteristics of fresh kiwifruit varieties.

Quality Characteristic	Varieties			
	Hayward	İlk Altın	HO8	J284
L*	77.21a	64.78bc	69.29b	58.85c
a*	−10.05c	−2.67ab	−3.56b	−1.71a
b*	20.11d	35.22a	27.55c	30.90b
TSS (%brix)	13.40c	14.4b	15.7a	14.86b
Acidity (%)	1.27a	0.88 cd	0.98c	1.12b
PH	3.45c	4.01b	4.24a	4.31a
Firmness (*N*)	9.43a	2.05d	2.97c	4.24b

*Note:* The lowercase letter indicates a statistically significant difference of at least 0.05 between the applications.

Some quality data for the fresh Hayward variety presented in Table 2 are taken from our previous publication (Göksel et al. 2022) and are used here as a green‐fleshed control reference.

**TABLE 2:** Physicochemical contents of fresh kiwifruit varieties (dry matter).

Varieties	Vitamin C (L‐ascorbik asit mg/100 g)	Total sugar (glucose + fructose mg/100 g)	Total phenol (GEDmg/100 g)	Antioxidant activity (ABTS) (TEDμmol/100 g)	Antioxidant activity (DPPH) (TEDμmol/100 g)	Antioxidant activity (FRAP) (TEDμmol/100 g)
**İlk Altın**	937.78a ± 50.47	98.67a ± 5.16	264.70b ± 28.29	1580.63b ± 150.87	171.09b ± 12.84	196.73b ± 12.24
**J284**	848.15b ± 39.02	72.08b ± 10.67	191.43c ± 38.03	711.90c ± 8.25	127.34c ± 14.34	153.02c ± 17.79
**HO8**	446.67c ± 35.12	61.46b ± 2.98	190.49c ± 10.08	817.14c ± 57.19	117.68c ± 6.44	138.79c ± 10.20
**Hayward**	433.33c ± 13.89	27.27c ± 1.26	541.39a ± 7.95	3895.65a ± 46.02	213.01a ± 8.01	232.03a ± 0.36
	*CV %5.56* *LSD 69.79*	*CV %8.61* *LSD 11.55*	*CV %8.26* *LSD 46.21*	*CV %4.79* *LSD 58.14*	*CV % 6.94* *LSD 20.53*	*CV %6.62* *LSD 22.43*

*Note:* The lowercase letter indicates a statistically significant difference of at least 0.05 between the applications.

### 2.11 Total Sugar Analysis

To determine the total sugar content, refractometric total sugar determination was performed according to the method used by Göksel et al. (2022), and the results were given in mg/100g.

### 2.12 Total Phenolic Contents

Total phenolic content and antioxidant activity (using three different methods) were analyzed in fresh and dried fruits of kiwifruit varieties. The Folin–Ciocalteu method was used to determine the total phenolic content of the samples, with minor modifications, in three replicates (Singleton and Rossi 1965). Briefly, 30 μL of extract and 150 μL of FC reagent were consecutively transferred into tubes containing 2.37 mL of deionized water. After 8 min, 450 μL of saturated Na_2_CO_3_ was added to the mixture. The same procedure was run to prepare the blank using 30 mL of deionized water instead of the extract. Absorbance values were read at 750 nm, using a UV–vis spectrophotometer (1–0003 model, U‐29000 HITACHI Instruments, Tokyo, Japan) against the blank, after 30 min incubation at 40°C. Various concentrations of gallic acid solutions (50, 100, 200, 300, 400, and 500 mg/L) were used to plot a calibration curve. The results are expressed as mg of gallic acid/mL (GAE mg/100 g fresh weight [FW]).

### 2.13 Total Antioxidant Activity

Three different methods were used to determine the antioxidant activity of kiwi samples and to compare the results of different methods. The extraction of the samples was performed according to the method used by Thaipong et al. (2006).

#### 2.13.1 DPPH (2,2‐Diphenyl‐1‐Picrylhydrazyl)

The stock solution was prepared by dissolving 24 mg DPPH with 100 mL methanol and then stored at −20°C until needed. The working solution was obtained by mixing 10 mL stock solution with 45 mL methanol to obtain an absorbance of 1.1 ± 0.02 units at 515 nm using the spectrophotometer. Fruit extracts (150 μL) were allowed to react with 2850 μL of the DPPH solution for 24 h in the dark. Then the absorbance was taken at 515 nm. The standard curve was linear between 25 and 800 μM Trolox. Results are expressed in μM TE/100 g dry mass. Additional dilution was needed if the DPPH value measured was over the linear range of the standard curve.

#### 2.13.2 Frap

The FRAP (Ferric Reducing Antioxidant Power) stock solutions included 300 mM acetate buffer (3.1 g C_2_H_3_NaO_2_·3H_2_O and 16 mL C_2_H_4_O_2_), pH 3.6, 10 mM TPTZ (2, 4, 6‐tripyridyl‐s‐triazine) solution in 40 mM HCl, and 20 mM FeCl_3_·6H_2_O solution. The fresh working solution was prepared by mixing 25 mL acetate buffer, 2.5 mL TPTZ solution, and 2.5 mL FeCl_3_·6H_2_O solution and then warmed at 37°C before using. Fruit extracts (150 μL) were allowed to react with 2850 μL of the FRAP solution for 30 min in the dark condition. Readings of the colored product [ferrous tripyridyltriazine complex] were then taken at 593 nm. The standard curve was linear between 25 and 800 μM Trolox. Results are expressed in μM TE/100 g dry mass. Additional dilution was needed if the FRAP value measured was over the linear range of the standard curve.

### 2.14 ABTS Radical‐Scavenging Activity

The stock solutions included 7.4 mM ABTSradical dot+ solution and 2.6 mM potassium persulfate solution. The working solution was then prepared by mixing the two stock solutions in equal quantities and allowing them to react for 12 h at room temperature in the dark. The solution was then diluted by mixing 1 mL ABTSradical dot+ solution with 60 mL methanol to obtain an absorbance of 1.1 ± 0.02 units at 734 nm using the spectrophotometer. Fresh ABTSradical dot+ solution was prepared for each assay. Fruit extracts (150 μL) were allowed to react with 2850 μL of the ABTSradical dot+ solution for 2 h in a dark condition. Then the absorbance was taken at 734 nm using the spectrophotometer. The standard curve was linear between 25 and 600 μM Trolox. Results are expressed in μM Trolox equivalents (TE)/100 g dry mass. Additional dilution was needed if the ABTS value measured was over the linear range of the standard curve.

### 2.15 Microbiological Analysis

Microbiological analysis: Bacterial and yeast‐mold counts were performed for the microbiological analysis of the dried kiwi slices. Total mesophilic aerobic bacteria and total yeast‐mold counts were determined by precise analysis. The counts were carried out using the method developed by Demirbüker et al. (2005).

### 2.16 Sensory Analysis

The method used by Göksel et al. (2022) was used in the sensory test analyses. Ethical approval for this sensory evaluation was granted by the Yalova Atatürk Horticultural Central Research Institute. All participants provided written informed consent prior to the study. Participants were informed of their right to withdraw at any time without penalty, and all food samples were prepared under strict safety and hygienic conditions. During the sensory analysis of kiwifruit slices, the following scale was used to score: 1: don't like. 2: very little like. 3: neither like nor dislike it. 4: little like. 5: a lot like. Sensory analysis was performed by around ten trained experts on dried slices of all kiwifruit varieties immediately after the drying process. During the sensory analysis on dried kiwifruits: color appearance (yellowish color‐bright green color), odor (excessively caramelized odor‐fresh, raw odor), texture (brittle‐elastic), and taste (fruit‐specific flavor) features were evaluated by the panelists.

### 2.17 Statistical Analysis

A randomized block design was used to statistically evaluate the effects of different kiwifruit varieties. Three replicates were used in all experiments, and the data were analyzed using analysis of variance. In addition, LSD tests were performed to examine significant differences between the means of the factors. Correlations between the data were calculated using a Pearson correlation coefficient of 0.05 (*p* = 0.05).

## 3 Results

In the analyses, the widely cultivated green‐fleshed *A. deliciosa* cv. Hayward variety was used as a control to compare basic quality characteristics of the fresh fruits of the three yellow‐fleshed varieties before drying. According to the results of the analysis, the highest value in terms of L* color was obtained in Hayward (77.21) and the lowest in J284 (58.85). While negative values were obtained in terms of another color value, a*, the highest was obtained from J284 (−1.71) and the lowest from Hayward (10.05). Regarding another color value, b*, the highest value was obtained from İlk Altın (35.22) and the lowest from Hayward (20.11). As expected, the highest value in Total Soluble Solids (TSS/Brix) was obtained from HO8 (15.7) and the lowest from Hayward (13.40) varieties. In terms of acidity, the highest value was obtained from the Hayward variety, as expected. In terms of pH value, yellow‐fleshed varieties gave higher values. Regarding fruit flesh hardness, the Hayward variety was much more complex than yellow‐fleshed varieties (Table 1).

In the analyses made in terms of vitamin C, it was determined that the yellow‐fleshed İlk Altın variety had the highest value and the Hayward variety had the lowest value. Similar results were obtained in terms of sugar with vitamin C. In terms of total phenol and antioxidant activity (three different methods), the highest values were found in the Hayward variety and the lowest values were obtained from the J 284 variety (Table 2).

In the color evaluations made on dried kiwifruit slices, it was determined that there was deterioration and darkening in the color of the fruits, especially after the 9th month. The highest loss in L* value was obtained in the İlk Altın (60.34) and J284 (59.13) varieties in the first month of storage, while the lowest value was obtained in the HO8 variety (39.42) in the 9th month of storage (Table 3).

**TABLE 3:** L* color values of dried yellow kiwifruit slices.

Varieties	Storage period (Month)				
	0	3	6	9	12
**İlk Altın**	60.34a ± 0.44	51.01e ± 0.69	42.67 h ± 0.88	49.69 fg ± 0.51	51.86e ± 0.24
**HO8**	56.32b ± 0.45	49.74 fg ± 0.39	48.87 g ± 0.13	39.42ı ± 1.67	50.87ef ± 1.45
**J284**	59.13a ± 0.75	51.04e ± 0.11	48.70 h ± 0.62	54.78c ± 0.57	53.12d ± 0.31

*Note:* CV % 1.47 LSD0.56. The lowercase letter indicates a statistically significant difference of at least 0.05 between the applications.

The highest in color a* value was obtained in the İlk Altın (6.49 and 6.11) variety the 9th and 12th months of storage, while the lowest value was obtained in the İlk Altın (1.27) and J284 (1.35) varieties (39.42) in the 1st month of storage (Table 4).

**TABLE 4:** a* Color values of dried yellow kiwifruit slices.

Varieties	Storage period (Month)				
	0	3	6	9	12
İlk Altın	1.27 h ± 0.11	5.89b ± 0.24	6.02b ± 0.67	6.11a ± 0.19	6.49a ± 0.27
HO8	3.43 g ± 0.079	4.09f ± 0.13	4.47def ± 0.13	4.85cde ± 0.27	5.01c ± 0.07
J284	1.35 h ± 0.15	4.45ef ± 0.45	4.89 cd ± 0.095	4.83cde ± 0.085	4.98c ± 0.05

*Note:* CV % 5.73 LSD 0.19. The lowercase letter indicates a statistically significant difference of at least 0.05 between the applications.

The highest in color b* value was obtained in the İlk Altın (39.55) variety, in the 12th month of storage, while the lowest value was obtained in the HO8 (26.29) variety (39.42) in the 3rd month of storage (Table 5).

**TABLE 5:** b* color values of dried yellow kiwifruit slices.

Varieties	Storage period (Month)				
	0	3	6	9	12
İlk Altın	36.19c ± 0.32	38.84ab ± 0.42	34.02d ± 0.25	38.94ab ± 0.15	39.55a ± 0.50
HO8	26.57j ± 0.34	26.29j ± 0.1	27.63ı ± 0.29	29.22 h ± 0.49	32.79e ± 1.11
J284	35.78c ± 0.07	30.36 g ± 0.23	31.32f ± 0.14	34.05d ± 0.07	34.12d ± 0.11

*Note:* CV %1.12 LSD 0.36. The lowercase letter indicates a statistically significant difference of at least 0.05 between the applications.

In the analyses made in terms of total antioxidant activity with ABTS method, the highest antioxidant activity value was obtained in dried slices of the İlk Altın variety (1140.16) at the end of 9 months of storage. The lowest antioxidant activity value was obtained in dried slices of the J284 kiwifruit variety (587.24) at the end of the first month (Table 6).

**TABLE 6:** Total antioxidant activity (TED μmol/100 g DM) of dried yellow kiwifruit slices by ABTS method.

Varieties	Storage period (Month)				
	0	3	6	9	12
İlk Altın	1083.75b ± 6.95	1087.29b ± 4.54	1048.41c ± 25.17	1140.16a ± 21.41	1099.41b ± 21
HO8	675.20f ± 4.62	687.95f ± 13.41	960.17d ± 17.91	647.87 g ± 21.98	685.25f ± 12.13
J284	587.24 h ± 7.55	680.83f ± 17.51	671.25 fg ± 6.61	718.31e ± 1.98	729.32e ± 12.97

*Note:* CV %1.78 LSD 13.56. The lowercase letter indicates a statistically significant difference of at least 0.05 between the applications.

In total antioxidant activity analyses performed with the DPPH method, the highest value was obtained in dried slices of the İlk Altın variety (89.87) stored for 1 month, while the lowest antioxidant activity value was obtained in dried slices of the J284 kiwifruit variety (53.74) at the end of the 12th month (Table 7).

**TABLE 7:** Total antioxidant activity (TED μmol/100 g DM) of dried yellow kiwifruit slices by DPPH method.

Varieties	Storage period (Month)				
	0	3	6	9	12
İlk Altın	89.87a ± 2.1	82.74b ± 1.4	70.25d ± 1.2	66.74ef ± 0.3	58.02ı ± 1.7
HO8	78.91c ± 2.9	70.57d ± 0.9	64.67 fg ± 1.8	62.63gh ± 0.5	57.06ı ± 1.8
J284	68.64de ± 0.1	64.66 fg ± 1.1	62.01 h ± 0.5	56.34ı ± 1.1	53.74j ± 0.62

*Note:* CV % 2.09 LSD 1.04. The lowercase letter indicates a statistically significant difference of at least 0.05 between the applications.

In the total antioxidant activity analyses performed by the FRAP method, the highest values were obtained from the dried slices of İlk Altın (107.55 and 105.89) and J284 kiwifruit varieties (107.14 and 106.62) stored for 1 and 12 months, while the lowest values were obtained from the dried slices of J284 kiwifruit variety (92.10) stored for 6 months (Table 8).

**TABLE 8:** Total antioxidant activity (TED μmol/100 g DM) of dried yellow kiwifruit slices by FRAP method.

Varieties	Storage period (Month)				
	0	3	6	9	12
İlk Altın	105.89a ± 4.87	93.29de ± 6.35	98.23c ± 1.09	96.15cde ± 2.98	107.55a ± 4.42
HO8	98.64bcd ± 6.23	105.0ab ± 7.41	94.09de ± 0.17	95.83cde ± 4.73	101.89abc ± 1.64
J284	106.62a ± 3.11	94.52de ± 1.01	91.51e ± 1.49	92.10de ± 3.67	107.14a ± 2.19

*Note:* CV %4.62 LSD2.08. The lowercase letter indicates a statistically significant difference of at least 0.05 between the applications.

In the analysis of total phenolic content in terms of Gallic Acid equivalent on dry matter, the highest value was obtained from dried slices of kiwifruit varieties İlk Altın (136.77) stored for 1 month and HO8 (132.03) and J284 (131.58) stored for 12 months. The lowest values were obtained from dried slices of İlk Altın variety stored for nine (100.96) and 12 months (100.21) and HO8 variety stored for 9 months (100.13) (Table 9).

**TABLE 9:** Total phenolic content (GAD mg/100 g DW) of dried yellow kiwifruit slices.

Varieties	Storage period (Month)				
	0	3	6	9	12
İlk Altın	136.77a ± 8.46	130.24ab ± 2.11	103.68e ± 1.87	100.96e ± 1.53	100.21e ± 3.84
HO8	129.49ab ± 5.4	129.29ab ± 11.13	122.58bc ± 3.23	100.13e ± 7.09	132.03a ± 0.18
J284	115.29 cd ± 2.98	113.24d ± 2.47	115.31 cd ± 2.05	116.96 cd ± 3.37	131.58a ± 5.97

*Note:* CV %4.25 LSD 3.73. The lowercase letter indicates a statistically significant difference of at least 0.05 between the applications.

The vitamin C content of dried yellow kiwifruit slices was determined as L‐ascorbic acid from the dry matter. The highest vitamin C value was obtained from dried kiwifruit slices of the J284 variety, which has yellow flesh and a red ring around the core. The lowest values were obtained from dried slices of the HO8 variety, especially those stored for 6, 9, and 12 months. In general, the vitamin C content of the HO8 variety showed lower values than the other two kiwifruit varieties (Table 10).

**TABLE 10:** Vitamin C content of dried yellow kiwifruit slices (L ascorbic acid mg/100 g DW).

Varieties	Storage period (Month)				
	0	3	6	9	12
İlk Altın	364.17b ± 15.06	363.33b ± 1.44	346.67cde ± 4.02	337.51e ± 5.0	239.16f ± 3.8
HO8	177.92 g ± 1.91	165.01gh ± 8.66	157.5 h ± 5.0	161.67 h ± 1.4	156.25 h ± 5.0
J284	395.12a ± 14.64	356.91bcd ± 3.73	360.16bc ± 15.9	340.65e ± 11.5	343.49de ± 3.72

*Note:* CV %4.62 LSD 6.20. The lowercase letter indicates a statistically significant difference of at least 0.05 between the applications.

Total sugar content was determined as the sum of fructose and glucose on dry matter. In the analyses, the highest total sugar value was obtained in dried slices of İlk Altın kiwifruit variety at the end of one (46.57) and 6 months (44.14) of storage. The lowest antioxidant activity value was obtained in dried slices of HO8 (18.70) and J284 (20.97) kiwifruit varieties stored for 12 months (18.70). Probably due to the deterioration that started at the end of the 9th month, serious decreases were observed in sugar content, especially in dry slices of HO and J284 varieties in the 12th month (Table 11).

**TABLE 11:** Total sugar content of dried yellow kiwifruit slices (Fructose+glucose mg/100 g DW).

Varieties	Storage period (Month)				
	0	3	6	9	12
İlk Altın	46.57a ± 0.98	43.18ab ± 4.12	44.14a ± 1.49	39.11c ± 1.83	37.47 cd ± 1.05
HO8	35.49d ± 3.07	31.05e ± 3.18	28.88ef ± 1.05	26.55f ± 0.85	18.70 g ± 2.81
J284	30.72e ± 1.17	28.82ef ± 0.33	39.74bc ± 2.25	31.93e ± 1.97	20.97 g ± 1.07

*Note:* CV %6.02 LSD 1.62. The lowercase letter indicates a statistically significant difference of at least 0.05 between the applications.

Losses (changes) between fresh fruit and dried fruit are given in percentages in Table 12. The highest loss in vitamin C was in the İlk Altın (61.17) variety, while the least loss was in HO8 (53.41). This situation clearly shows that vitamin C is lost by more than half during drying, especially in yellow‐fleshed varieties. Similarly, the highest loss in total sugar loss was detected in the İlk Altın variety (62.10), while the lowest loss was detected in the J 284 variety (42.60). Losses in terms of total phenol were slightly more limited. The highest loss was detected in the İlk Altın variety (48.33) again, while the lowest loss was detected in the J284 variety (32.02). In terms of total antioxidant, the highest loss was obtained from the İlk Altın variety in all methods. The varieties with the least antioxidant loss were the HO8 variety in the FRAP method, and the J28 variety in the ABTS and DPPH methods.

**TABLE 12:** Loss values in fresh and dried kiwifruit varieties (%).

Varieties	L‐ascorbic acid (Vitamin C)	Sugar (Total)	Phenol (total)	Total antioxidants		
				ABTS method	DPPH method	FRAP method
İlk Altın	61.17	62.10	48.33	31.00	47.51	46.17
J284	60.17	42.60	32.02	5.00	38.03	35.54
HO8	53.41	57.30	39.77	28.00	41.67	23.18

When the sensory analysis results of dried kiwifruit slices are examined, it is seen that they were liked by the panelists by receiving very high scores. In the evaluation made out of 25 points, the averages of the scores given by the panelists were taken. According to the evaluation results, especially the HO8 and J284 kiwifruit varieties were liked slightly more than the İlk Altın variety (Table 13).

**TABLE 13:** Sensory analysis results of dried kiwifruit slices based on varieties (1: Don't like. 2: Very little like. 3: Neither like nor dislike it. 4: Little like. 5: A lot like).

Sensory characteristic	Varieties		
	HO8	J284	İlk altin
View‐Color	5	5	4
Smell	5	5	5
Texture	4	4	4
Taste	5	5	5
Overview	5	5	5
TOTAL	24	24	23

In all kiwifruit varieties, water activity values were below 0.60 aw during the initial storage periods, and therefore, yeast‐mold and total viable microorganism growth was not observed. Changes in the varieties during storage were evaluated alongside the microbiological results. The aw values of the kiwifruit varieties during storage are as follows:

- 0th month: J284 0.50; İlk Altın 0.55; HO8 0.52.
- 3th month: J284 0.52; İlk Altın 0.52; HO8 0.56.
- 6th month: J284 0.56; İlk Altın 0.53; HO8 0.56.
- 9th month: J284 0.56; İlk Altın 0.54; HO8 0.54.
- 12th month: J284 0.48; İlk Altın 0.45; HO8 0.53.

The shelf life endpoint is based not only on microbial counts but also on a holistic assessment of microbiological safety, along with water activity, moisture content, color changes, and sensory acceptability criteria. In the total viable count and total mold yeast count analyses, no growth was observed in all varieties in the first 6 months. However, reproduction was detected in all varieties by the ninth month and increased significantly, especially in the twelfth month. These results show that yellow‐fleshed dried kiwifruit varieties can be stored up to 6 months, and that longer storage is not possible under current conditions. For longer storage, lower temperature conditions must be used (Table 14).

**TABLE 14:** Total viable count (cfu g^−1^) and total mold yeast count (cfu g^−1^) in kiwifruit slices during the storage period.

Storage period (Month)	Total viable count (cfu g^−1^)			Total mold yeast count (cfu g^−1^)		
	HO8	J284	İlk Altın	HO8	J284	İlk Altın
0	< 10	< 10	< 10	< 10	< 10	< 10
3	< 10	< 10	< 10	< 10	< 10	< 10
6	< 10	< 10	< 10	< 10	< 10	< 10
9	1.29 × 10^1^	7.01 × 10^1^	1.41 × 10^1^	2.71 × 10^1^	1.56 × 10^1^	3.96 × 10^1^
12	2.67 × 10^2^	2.40 × 10^2^	2.53 × 10^2^	4.48 × 10^2^	2.73 × 10^2^	4.73 × 10^2^

## 4 Discussion

The species that is widely grown in the world and can be stored for a long time is *Actinidia deliciosa* cv. Hayward, although in recent years consumers have shown more demand for new varieties with new meat color, taste and appearance in international markets. While green‐fleshed kiwifruit varieties can be stored in cold storage conditions for a long time, many yellow‐fleshed varieties can be stored for a much shorter time (Zhong et al. 2012). For this reason, drying kiwifruit alternative product processing techniques are very important for growers to sell their fruits at their value (Radojčin et al. 2021). In addition, it is the most correct approach to know the changes in important quality components of fresh and dried kiwifruits and recommend them to consumers with their final contents.

As seen in our study, some of the quality components in fresh fruits may be lost during drying, depending on the drying method used. This situation can be caused by multiple factors. Studies conducted by researchers (Huang et al. 2021) have shown a situation similar to our study. Many factors contribute to these differences. Among the most prominent are the ecology of the growing environment, the nutritional conditions of the trees, different cultivation techniques, and the methods preferred during the storage/ripening stages of different fruits.

In kiwifruit, especially dry matter content is considered a very important indicator of eating quality and is expressed as a percentage of fruit weight. Based on this importance, all results in our study were evaluated on dry matter. When we look at the TSS, pH, total sugar, acidity and vitamin C values obtained in the study, it is reported that they are similar to the studies conducted by different researchers (Krupa et al. 2011); Vincenzo et al. (2024).

Sivakumaran et al. (2018) reported that in the study they conducted on vitamin C in yellow‐fleshed kiwifruits, the SunGold variety found 109–161 mg/100 g vitamin C, while in the green‐fleshed Hayward variety, this rate was 85 mg/100 g. In our study, the highest value in terms of vitamin C was determined as 157 mg/100 g in the J284 variety. The reason why the amount of vitamin C is higher in this variety compared to others is thought to be the presence of red color pigments around the woody middle part. Both ecological differences and producer practices directly affect the fruit quality characteristics of the cultivated kiwifruit. While our study results show similarities in many aspects when compared to similar studies in the literature, some differences are also observed. The main reasons for these differences include ecological conditions, nutritional status, and different ripening methods used.

In addition, researchers reported that fructose is the highest in kiwifruit, sucrose is the lowest, and soluble solids in kiwifruit are mainly composed of glucose and fructose Monro (2013). Therefore, in this study, the total value of fructose and glucose was determined by analysis.

In their study, Mohammadi et al. (2008) stated that color loss can result from the decomposition of chlorophyll and carotenoid pigments, the non‐enzymatic Maillard reaction, and the formation of brown pigments. Furthermore, Diamante et al. (2010) and Kaya et al. (2010) also reported, similarly to our results, that vitamin C decreases (19%) and significant color loss occurs in kiwifruits during the drying process, especially due to temperature. The significant decrease in ascorbic acid levels in dried fruits during storage is attributed to spoilage. This is because ascorbic acid is a highly perishable vitamin and can be easily broken down under various conditions, such as pH and temperature changes, light, oxygen, enzymes, and the presence of metallic catalysts. Yılmaz and Ersus Bilek (2017) reported that significant changes in the bioactive components of various fruit and vegetable types can be detected during drying using different methods. Due to the heat treatments applied to different types of dried foods, levels of various phytochemicals, particularly antioxidant activity, can change. The results from our study on different kiwifruit varieties showed a trend similar to that reported in the literature (Orikasa et al. 2014). In fruits that contain many natural antioxidants, multiple methods are typically used to assess their antioxidant potential. In particular, the ABTS, DPPH, and FRAP methods we used are among the most commonly used analytical methods. The main reason is the complex structure of phytochemicals; researchers often analyze total antioxidant activity using multiple methods.

Orikasa et al. (2014) reported that vacuum‐ and hot‐air‐dried kiwifruit can cause significant changes in fruit color, vitamin C, and antioxidant activity, findings similar to ours. Similarly, Serrano et al. (2005) found that the total antioxidant content of kiwifruits was largely influenced by vitamin C and polyphenol levels, results consistent with our study.

Total Antioxidant Activity values decreased until the ninth month during storage, while an increase was observed in the twelfth month. The high antioxidant activity level at the beginning of storage is explained by the chemical and enzymatic oxidation of phytochemical compounds. In addition, researchers reported that total antioxidants and compounds such as vitamin C, which are high in kiwifruits in the pre‐harvest period, decreased when fruit becomes overripe (Gullo et al. 2016).

As a result of drying fruits with different methods, different results can be obtained in the changes in the amounts of phenolic compounds and total antioxidants in their contents. The results obtained may differ, especially according to the dried species, variety, pretreatments applied, and drying method (Sultana et al. 2012).

Jesionkowska et al. (2009) reported that changes in taste and appearance can be observed in different fruits dried depending on certain conditions. These changes can include taste, aroma, texture, and even nutritional content. The main reason for this can be attributed to heat treatment and the resulting deterioration. However, although there are losses in vitamins such as vitamin C, there are also positive aspects in terms of mineral content, antioxidant activity, and energy values.

When determining the endpoint for shelf life, not only microbial counts but also water activity, moisture content, color changes, and sensory acceptability criteria, along with microbiological safety, were considered holistically. In light of these results, no yeast or mold formation was observed in any of the yellow‐fleshed kiwifruit varieties until the 6th month of storage. This may be related to the water activity being below 0.6 aw, as low water activity is reported to inhibit microbial growth. It is reported that this low range may be effective in long‐term storage of dried fruits (Aktaş and Kara 2013).

## 5 Conclusions

Kiwifruit production is rapidly increasing worldwide, especially with the use of new varieties developed through breeding programs. The increased production must be evaluated in different ways beyond fresh consumption. Drying technology has been widely used for this purpose in recent years. However, depending on the drying conditions, some changes may occur in the sensory and nutritional properties of dried products. In line with the results of our study, although heat treatment results in some losses in certain quality components, such as vitamin C, they meet expectations quite well, especially in terms of fiber and minerals, and can also be stored for a long time at low energy costs. Because the water content of kiwifruit is significantly reduced by drying, its quality characteristics have remained within the safe food range for a long time. Especially, the fact that yellow‐fleshed varieties largely retain their color after drying, along with high brix and low acidity, are the most important characteristics that will attract consumer attention. When the results of this study were evaluated, it was found that higher‐value‐added products can be obtained using the heat pump drying method. Further studies focusing on reducing losses in quality components through different new drying methods, as well as examining their shelf life, could yield more detailed results.

## Author Contributions

**A. Atak:** conceptualization, investigation, writing – original draft, writing – review and editing. **K. A. Kahraman:** conceptualization. **C. Tunçkal:** methodology, formal analysis. **Z. Göksel:** data curation, formal analysis, investigation, methodology.

## Funding

This study was supported by the General Directorate of Agricultural Research and Policies (TAGEM/HSGYAD/E/18/A3/P3/378).

## Disclosure

AI Use Statement: The authors did not use generative AI tools in the preparation of this manuscript.

## Conflicts of Interest

The authors declare no conflicts of interest.

## Data Availability

The data that support the findings of this study are available from the corresponding author upon reasonable request.
